# Supplementary material for: The structural basis of tRNA recognition by arginyl-tRNA-protein transferase
Source: Nat Commun. 2023 Apr 19;14:2232. doi: 10.1038/s41467-023-38004-8 (PMC10115844; doi:10.1038/s41467-023-38004-8)
Supplement: Supplementary file 1 — Supplementary Information [file 41467_2023_38004_MOESM1_ESM.pdf]

Supplementary Figure 1

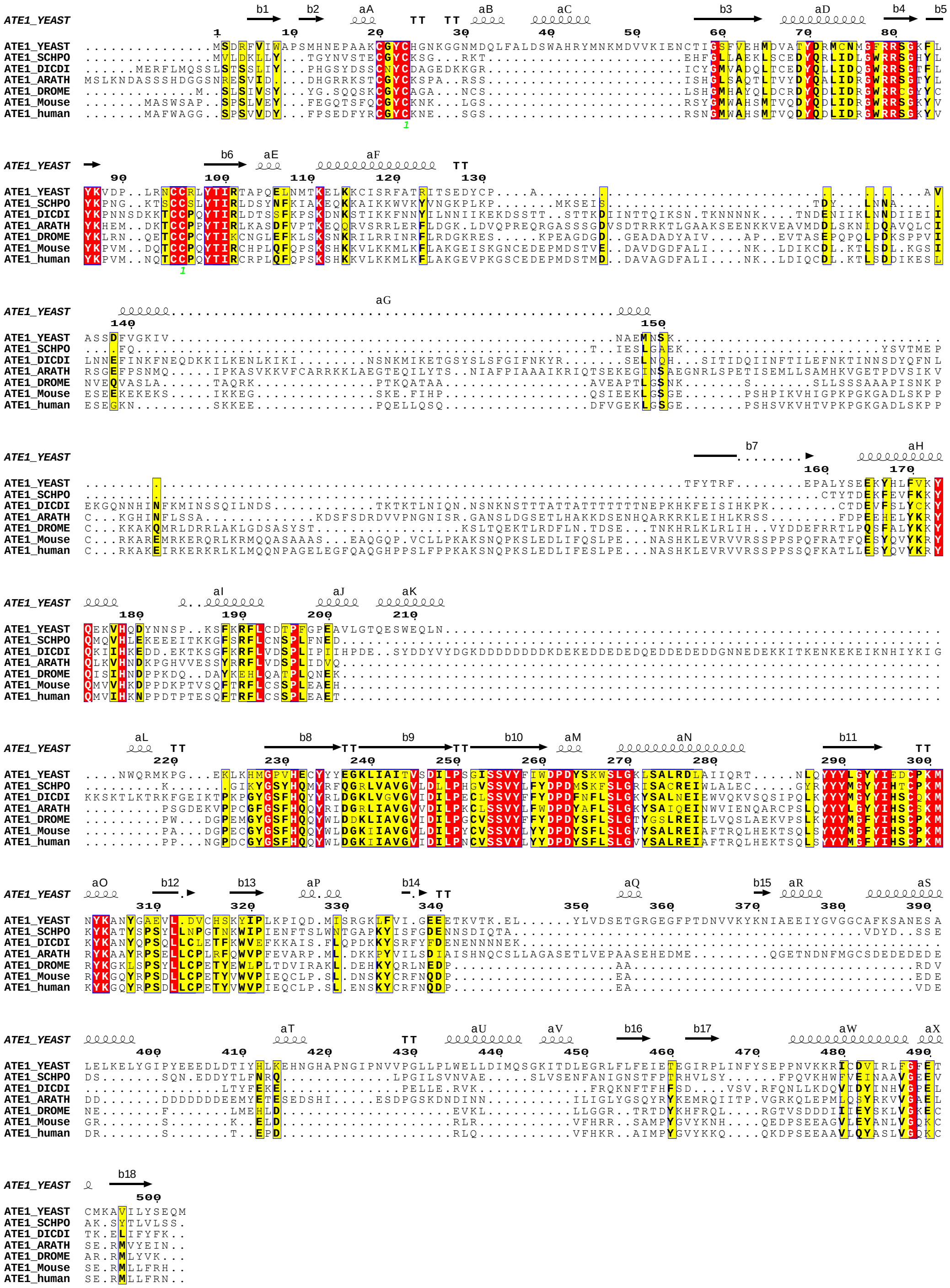

Supplementary Figure 1. Multiple sequence alignment of ATE1 sequences in Uniprot from model organisms (yeast, SCHPO, DICDI, ARATH, DROME, mouse, and human) using Clustal Omega and displayed using ESPript3. Arrows and spirals indicate β-strands and α-helices, respectively. Red and yellow backgrounds indicate identical and similar residue.

Supplementary Figure 2

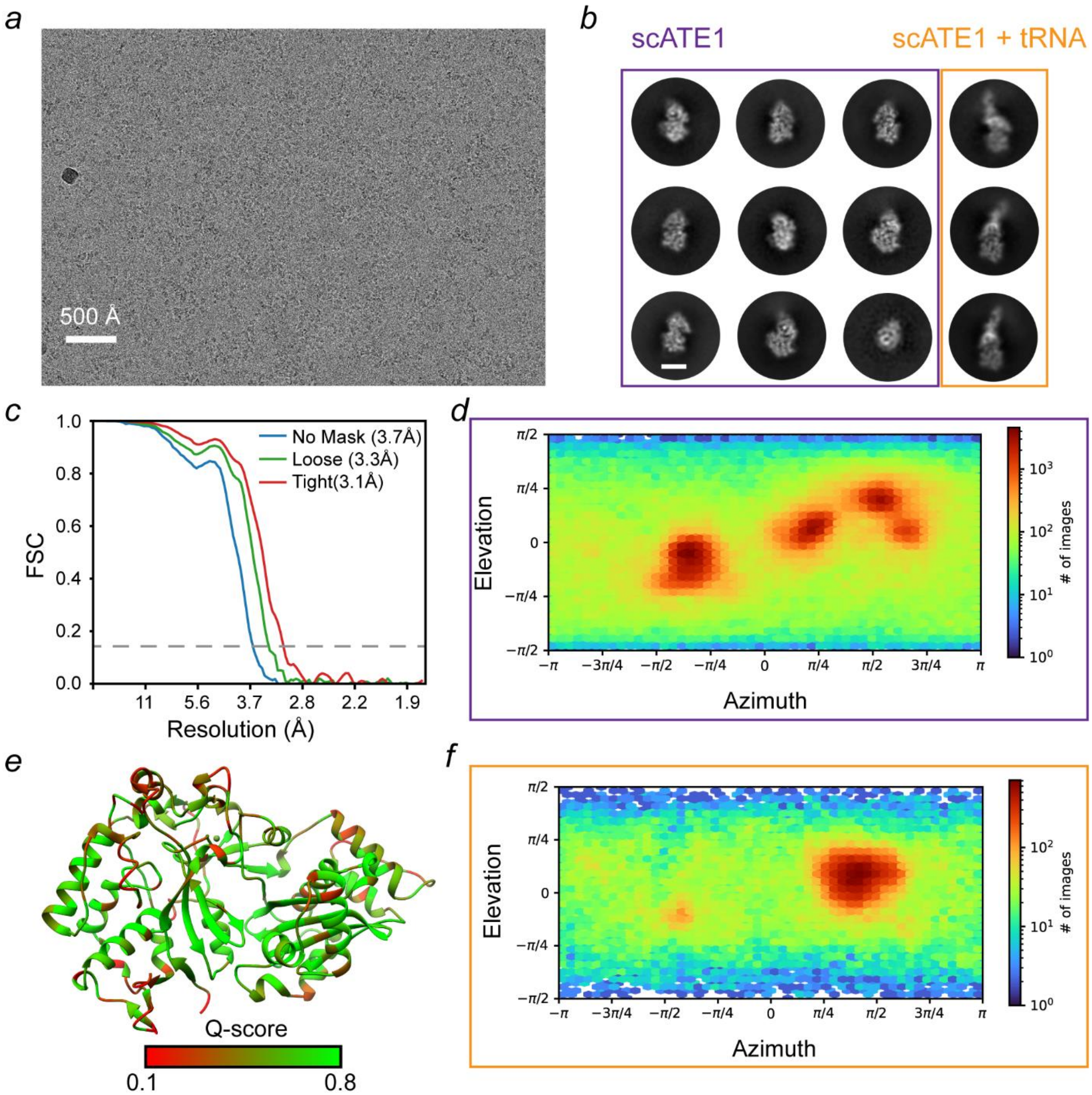

Supplementary Figure 2. CryoEM image processing. (a) A representative micrograph of *sc*ATE1 cryo-grids. The scale bar is 500 Å. (b) Representative 2D class averages. Apo *sc*ATE1 particles are highlighted in purple box and *sc*ATE1-tRNA particles are in orange box. The scale bar is 50 Å. (c) FSC curve for 3D reconstruction maps of apo *sc*ATE1. The grey dash line indicates the 0.143 FSC cutoff. (d) Angular distribution of apo *sc*ATE1 particle set. (e) Q-scores, representation the resolvability of the structure model from the cryoEM map, of individual residues colored on *sc*ATE1 structure. Majority of residues, except these in the loop and on the protein surface, have a high Q-score. (f) Angular distribution of *sc*ATE1-tRNA particle set.

Supplementary Figure 3

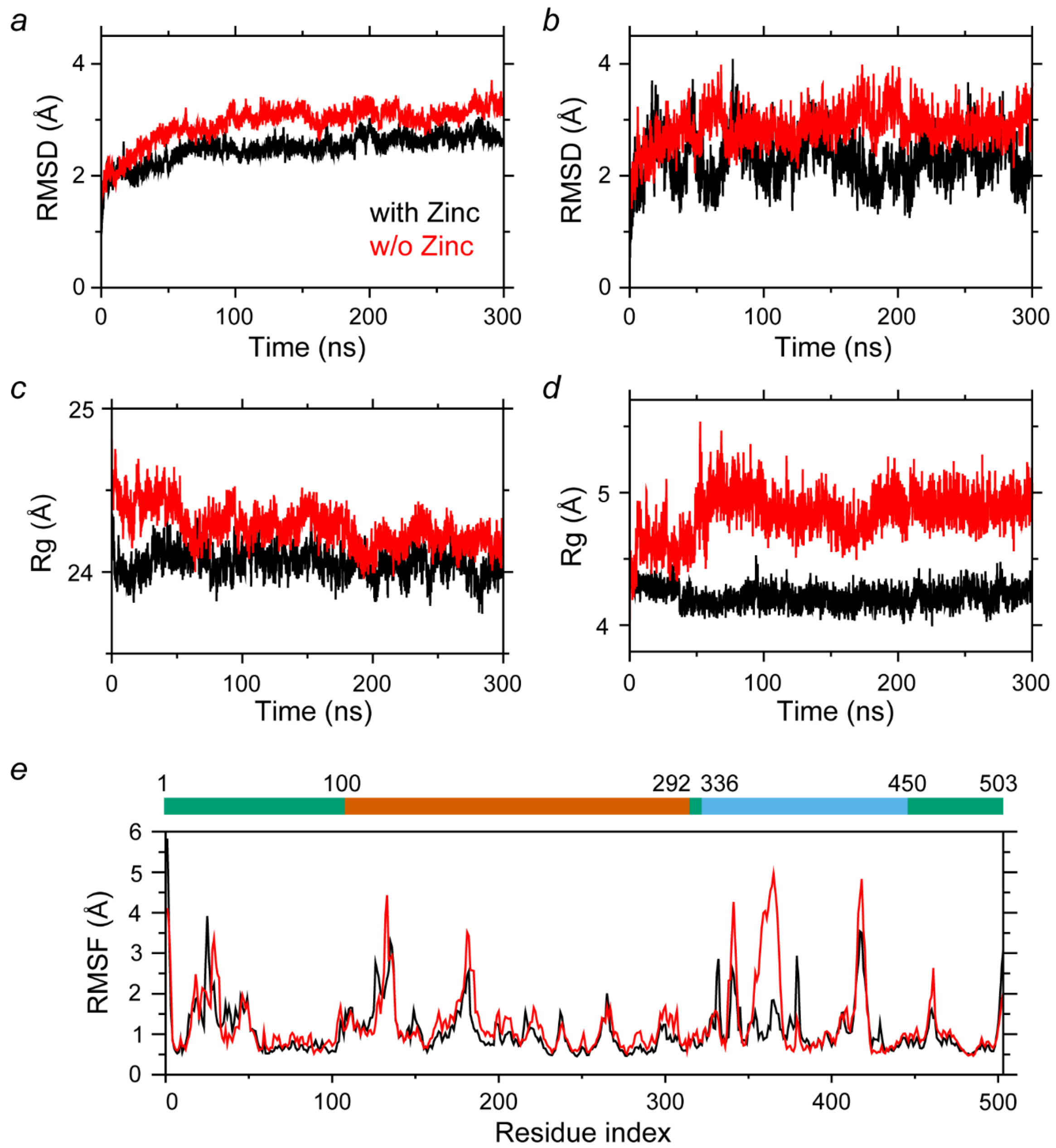

Supplementary Figure 3. MD simulation of *sc*ATE1 with and without Zinc. (a) Time evolution of root-mean-square-deviation (RMSD) for *sc*ATE1 with (black line) and without (red line) zinc with reference to the cryoEM structure. (b) Time evolution of RMSD for four cysteines (Cys 20, 23, 94 and 95) with (black line) and without (red line) zinc with reference to the cryoEM structure. (c) Radius of gyration of the whole scATE1 protein. (d) Radius of gyration of the four-cysteine cluster. (e) Root-mean-square-fluctuation (RMSF) of *sc*ATE1 with (black line) and without (red line) Zinc. Domain organization of scATE1 was plot on the top of the panel.

Supplementary Figure 4

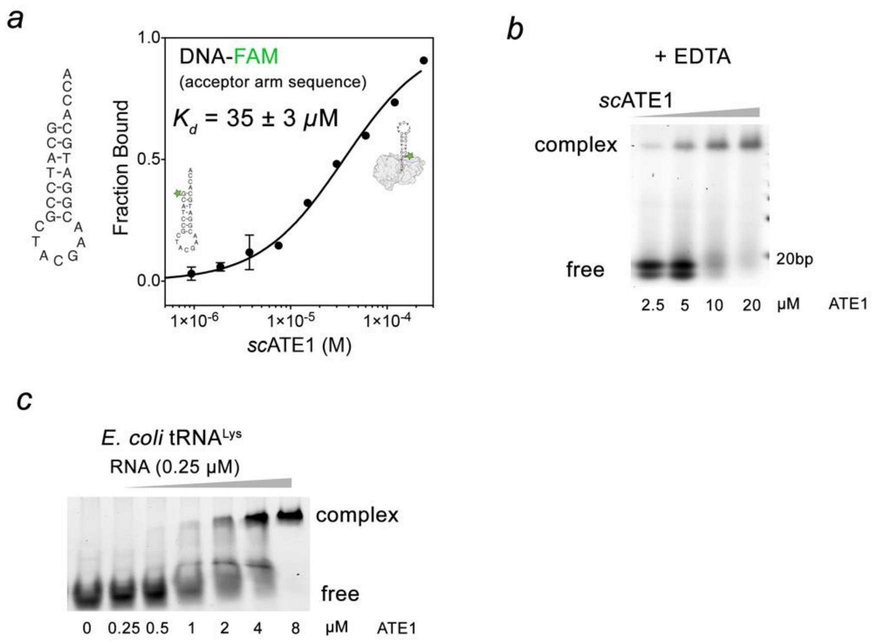

Supplementary Figure 4. ATE1-ZnF is critical for RNA binding. (a) Binding curves used to determine the K_d value of ATE1-DNA interaction in a direct binding assay by MST. Error bar represents s.d. in duplicate measurements. (b) EMSA visualization of ATE1 binding to synthesized RNA in the presence of 10 mM EDTA. ATE1 concentrations were shown below the gel. Two independent experiments were performed with similar results. (c) Representative EMSA assay of ATE1 binding to *in vitro* transcribed $tRNA^{Lys}$. Three independent experiments were performed with similar results.

Supplementary Table 1. Data collection and refinement statics for the *sc*ATE1.

	*sc*ATE1	*sc*ATE1 + $tRNA^{Arg}$
Microscope	Titan Krios	
Voltage (keV)	300	
Image filter	BioQuantum	
Slit width (eV)	20	
Super-resolution Pixel size (Å)	0.4363	
Symmetry	C1	
Dofocus range (micron)	-0.5 to -2.5	
Electron dose ($e^-/Å^2$)	40	
Micrographs	13,455	
Number of particles	523,915	208,051
Map resolution at 0.143 FSC (Å)	3.1	3.6
B-factor	152.7	156
Model Refinement		
Atom	4,072	5711
Residues	Protein: 503, Nucleotides: 0	Protein: 503, Nucleotides: 77
Ligands	ZN: 1	ZN: 1
CCmask	0.79	0.51
Resolution (FSC map vs. model at 0.5) (Å)	3.8	4.1
r.m.s. deviations		
Bond lengths (Å)	0.003	0.002
Bond angles (°)	0.594	0.450
Clash score	8.81	10.73
MolProbity score	1.76	2.49
PDB ID	8E3S	8FZR
EMDB ID	27871	29638
