# Supplementary material for: The structural basis of tRNA recognition by arginyl-tRNA-protein transferase
Source: Nat Commun. 2023 Apr 19;14:2232. doi: 10.1038/s41467-023-38004-8 (PMC10115844; doi:10.1038/s41467-023-38004-8)
Supplement: Supplementary file 3 — Description of Additional Supplementary Files [file 41467_2023_38004_MOESM3_ESM.pdf]

**Description of Additional Supplementary Files**

File Name: Supplementary Movie 1

Description: Conformational change of scATE1 upon tRNA binding. Morphing of two scATE1 structure models between the apo form and the tRNA bound state. The movie starts with the apo-form transiting to the tRNA bound conformational state. GNAT domain is shown in orange, substrate binding domain and variable domain are shown in green. The tRNA structure model is shown at the 2nd half of the movie in purple. An arginine amino acid, show in blue, was docked into the cavity suggested by the blL-PGS and ecLFTR structures (PDB entries: 4v36 and 2z3k).
